# SOC estimation of lead–carbon battery based on GA-MIUKF algorithm

**DOI:** 10.1038/s41598-024-53370-z

**Published:** 2024-02-09

**Authors:** Lu Wang, Feng Wang, Liju Xu, Wei Li, Junfeng Tang, Yanyan Wang

**Affiliations:** School of Machinery and Transportation, Southwest Forestry University, Kunming, 650224 China

**Keywords:** Electrical and electronic engineering, Energy

## Abstract

The paper proposes a SOC (State of Charge) estimation method for lead–carbon batteries based on the GA-MIUKF algorithm. The GA-MIUKF algorithm combines GA (Genetic Algorithm) for global search and optimization with the MI-UKF (Multi-innovation Unscented Kalman Filter) algorithm for estimating the SOC of lead–carbon batteries. By establishing an equivalent circuit model for the battery, the GA is employed to globally search and optimize the battery model parameters and the noise variance parameters in the MI-UKF algorithm. Comparative analyses with the UKF (Unscented Kalman Filter) algorithms and MI-UKF algorithms reveal that the SOC estimation method based on the GA-MIUKF algorithm yields more accurate results for lead–carbon battery SOC estimation, with an average estimation error of 2.0%. This highlights the efficacy of the proposed approach in enhancing SOC estimation precision.

## Introduction

Lead–carbon batteries, as a mature battery technology, possess advantages such as low cost, high performance, and long lifespan, leading to their widespread application in energy storage and power battery fields^1,2^. However, in practical engineering, lead–carbon batteries face challenges, such as significant SOC estimation errors, resulting in inaccurate estimations that directly impact the performance and reliability of these batteries.

Accurate SOC estimation for lead–carbon batteries is crucial for their daily management and maintenance. SOC is a vital parameter representing the remaining charge capacity of the battery^3^. Currently, common SOC estimation methods include open-circuit voltage method, ampere-hour integration method, and Kalman filtering method. He et al.^4^ proposed an online SOC estimation method for lithium-ion batteries based on the open-circuit voltage method. This method models and analyzes the dynamic evolution of SOC based on the relationship between the battery's open-circuit voltage (OCV) and SOC. It derives a recursive function for online identification of the battery OCV, establishes an accurate OCV–SOC lookup table, and achieves real-time estimation of SOC for lithium-ion batteries. Reference^5^ combines Extended Kalman Filtering (EKF) with current integration method to estimate the SOC of lithium-ion batteries. This method effectively reduces errors in current integration and inaccuracies in model identification. Dai et al.^6^ use a dual-time-scale Kalman filter to estimate the SOC of lithium-ion batteries online. This approach decouples SOC and capacity estimation from both measurement and time scale perspectives, significantly reducing the computation time required for obtaining SOC and capacity estimates and improving the accuracy and real-time performance of SOC estimation. Li et al.^7^ propose a SOC estimation method based on improved adaptive Unscented Kalman Filtering. This method effectively reduces the impact of model and measurement errors on estimation results, enhancing estimation accuracy and stability. Liu et al.^8^ introduce an improved adaptive Extended Kalman Filtering method, incorporating a feedforward compensation method to reduce errors in OCV identification. This enhancement improves the SOC estimation method based on the open-circuit voltage, effectively minimizing the impact of noise interference on SOC estimation and improving accuracy and stability.

In summary, existing SOC estimation methods for batteries mainly focus on open-circuit voltage, ampere-hour integration, and filtering algorithms. While these methods improve estimation accuracy and stability to some extent, they still face various challenges and issues related to battery operating conditions, battery model optimization, and computational resources. Therefore, this paper proposes a SOC estimation method based on the GA-MIUKF algorithm, utilizing genetic algorithms for global search and optimization of battery model parameters to estimate the SOC of lead–carbon batteries under complex current conditions such as the UDDS cycle. During the validation of the algorithm, a comparative analysis of estimation outcomes is conducted for the UKF and MIUKF algorithms under identical operating conditions, assessing the accuracy and practical applicability of the proposed methodology. The research findings presented in this paper contribute significantly to the ongoing enhancement of efficiency and management proficiency in lead–carbon batteries.

The paper is structured into five sections, outlined as follows:

- **Introduction:** Introduction to the Characteristics of Lead–Carbon Batteries and the Research Background, Significance, Objectives, Main Research Content, and Methodology of Battery State of Charge (SOC) Estimation.
- **Battery modeling:** The GNL circuit is chosen as the model for lead–carbon batteries, providing the foundational estimation for subsequent State of Charge assessments.
- **Methodology:** Details the GA-MIUKF method for estimating the SOC of lead–carbon batteries.
- **Results and Discussion:** Battery subjected to HPPC and simulated UDDS tests, combined with identified parameter results, compared the estimation results of UKF, MIUKF, GA-MIUKF algorithms under UDDS working conditions, and evaluated the accuracy and practicality of the proposed method.
- **Conclusion:** Summarizes the main research content and conclusions of the paper.

Through this structure, the paper aims to conduct an in-depth study and analysis of SOC estimation for lead–carbon batteries. It introduces the GA-MIUKF method for estimating the SOC of lead–carbon batteries and aims to provide robust support for research and applications in related fields.

## Battery modeling

**Figure 1:**
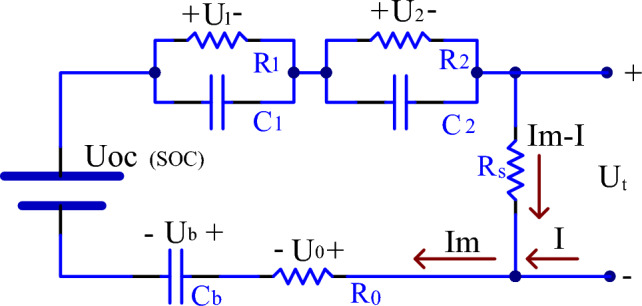
Second-order GNL circuit model of lead–carbon battery.

Lead–carbon batteries are commonly used in energy storage applications, and modeling their performance is a crucial area of research in battery management systems. The circuit equivalent model is one of the most commonly used methods in battery modeling, and the GNL (Gummel-Null Line) equivalent circuit is a specific circuit model employed to describe the behavior and performance of batteries during charge and discharge processes^9,10^. In the equivalent circuit, the charge and discharge processes of the battery can be represented by an RC circuit composed of internal resistance and electrochemical capacitance. During discharge, the electrochemical potential source indicates that the battery's potential decreases as the battery discharges. Conversely, during charging, the electrochemical potential source indicates that the potential of the charging power supply increases as the battery charges. The GNL equivalent circuit model can be utilized to predict the performance of lead–carbon batteries under various operating conditions. Figure 1 below illustrates a second-order GNL model as the equivalent model for lead–carbon batteries.

The following equations are constructed from the second-order GNL model:

1 $$U_{t} = U_{oc} - U_{1} - U_{2} - U_{b} - R_{0} I_{m}$$

2 $$U_{b} = \frac{{\int {I_{m} dt} }}{{C_{b} }}$$

3 $$I_{m} = \frac{{U_{1} }}{{R_{1} }} + C_{1} \frac{{dU_{1} }}{dt}$$

4 $$I_{m} = \frac{{U_{2} }}{{R_{2} }} + C_{2} \frac{{dU_{2} }}{dt}$$

5 $$U_{t} = (I_{m} - I)R_{s}$$

In this model, *U*_*t*_ represents the battery terminal voltage, *U*_*oc*_ is the static electromotive force of the battery, *I* denotes the charging and discharging current of the battery, *R*_0_ signifies the contact resistance and internal ohmic resistance of various processes within the battery cell, *R*_1_ and *C*_1_ represent the internal polarization phenomenon of the battery cell, *R*_2_ and *C*_2_ manifest the internal diffusion phenomenon of the battery, *C*_b_ stands for the equivalent capacitance of the battery, and *R*s represents the self-discharge resistance of the battery. The second-order GNL model can intricately simulate the internal chemical reactions and ion migration of the lead–carbon battery. In comparison to traditional circuit equivalent models, it can more accurately predict the battery's performance, thereby enhancing the precision and efficiency of battery management systems.

## Methodology

### Genetic algorithm for model parameters

**Figure 2:**
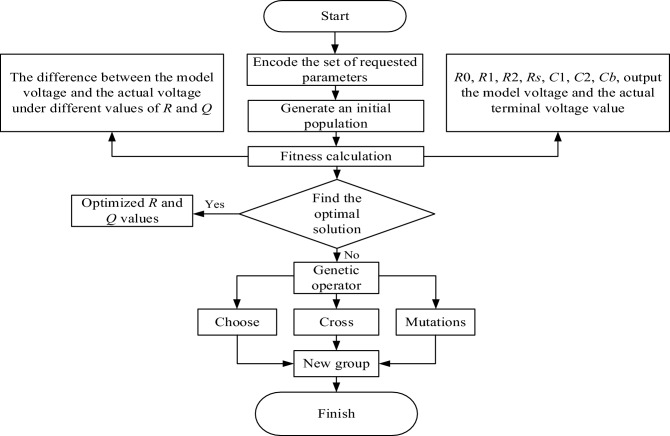
Genetic algo identification lead–carbon battery parameter flow chart.

The Genetic Algorithm is an optimization algorithm based on the principles of natural evolution and genetic mechanisms^11,12^. It simulates processes such as selection, crossover, and mutation that occur in biological evolution. By encoding and evolving candidate solutions, it continuously optimizes and evolves to find the optimal solution or an approximate optimal solution. The advantages of genetic algorithms lie in their ability to handle complex nonlinear problems, possess global optimization capabilities, and are not constrained by conditions or influenced by initial solutions. They can be applied to parameter optimization in the battery parameter identification process, specifically using optimization algorithms to search for the optimal parameter solution, thereby achieving improved parameter estimation, as illustrated in Fig. 2.

1. Encoding the set of system parameters:

In the process of battery parameter identification, the model parameters of lead-carbon batteries identified using the forgetting factor least squares method are encoded into binary. These encoded data will be used to assess the accuracy and reliability of battery parameter estimation.

2. Initializing the population:

Utilizing the data of battery model parameters, a set of initial parameter solutions is generated as the population. Each parameter solution constitutes a vector of battery model parameters, essentially forming chromosomes.

3. Fitness calculation:

Each individual's parameter solution is entered into the fitness function to calculate its fitness value, assessing its ability to solve the problem. The fitness function is typically defined by comparing the error between model-predicted values and actual measured values.

4. Selecting the optimal solution:

Through the selection operator, some excellent individuals are chosen from the current population as the parents of the next generation. Here, Q and R are covariance matrices used to describe the characteristics of noise in the system and measurement process. Q represents the covariance of system process noise, while R represents the covariance of measurement noise. The algorithm employs Q = 0.1 and R = 0.001, determined through a parameter tuning process. This process involves conducting sensitivity analysis and using genetic algorithms to optimize parameters to minimize estimation errors.

5. Crossover Operation:

Perform crossover operations on the parent individuals to generate new individuals. In battery parameter identification, single-point or multi-point crossover operators can be employed, involving the exchange of a portion of the chromosome in parent individuals.

6. Mutation Operation:

Apply mutation operations to the newly generated individuals to ensure population diversity, preventing convergence to local optima. In battery parameter identification, mutation operations may involve random changes at random positions in the chromosome.

7. Obtain a New Population:

Merge the newly generated individuals with the original population to form the new generation.

8. Termination Criteria:

Evaluate whether termination criteria are met, such as reaching the maximum iteration count or achieving a sufficiently excellent value for the objective function.

Repeat steps 4–8 until the termination conditions are met. Once the termination conditions are satisfied, output the optimal solution or optimal individual as the algorithm's result, representing the best-estimated parameters for the battery.

### MI-UKF algorithm

The MI-UKF algorithm is an improved version of the UKF algorithm^13^, utilized for state estimation in systems. Compared to traditional Extended Kalman Filter (EKF) and UKF algorithms, the MI-UKF algorithm can more accurately estimate the states of nonlinear systems. In the UKF algorithm, a set of Sigma points is chosen to approximate the probability distribution of state variables, facilitating state estimation in nonlinear systems. However, over time, the number of Sigma points increases rapidly, leading to an escalation in the computational complexity of the system. The MI-UKF algorithm addresses this issue by introducing a new information matrix. During each measurement, the MI-UKF algorithm utilizes this new information matrix to update the Sigma points, reducing their quantity and concurrently enhancing the accuracy of state estimation and computational efficiency^14^.

Steps in the application of the MI-UKF algorithm for estimating the State of Charge (SOC) in lead–carbon batteries:

1. Initialize the mean $\overline{x}_{0}$ of the battery state vector *X*0 and the state estimation error covariance matrix *P*0.

6 $$\left\{ \begin{gathered} \overline{x}_{0} = E(x_{0} ) \hfill \\ P_{0} = E[(x_{0} - \overline{x}_{0} )(x_{0} - \overline{x}_{0} )^{T} ] \hfill \\ \end{gathered} \right.$$

2. Calculating ***Sigma*** points at *k*-1 moments:

***Sigma*** points are calculated using the predicted state vector and covariance matrix to estimate the nonlinear distribution of states.

7 $$\left\{ \begin{gathered} x_{0,k - 1} = \overset{\lower0.5em\hbox{$\smash{\scriptscriptstyle\frown}$}}{x}_{k - 1} \hfill \\ x_{\iota ,k - 1} = x_{k - 1} + \left( {\sqrt {(n + \gamma )P_{k - 1} } } \right)_{\iota } ,\quad (\iota = 1, \ldots ,n) \hfill \\ x_{\iota ,k} = \overset{\lower0.5em\hbox{$\smash{\scriptscriptstyle\frown}$}}{x}_{k - 1} - \left( {\sqrt {(n + \gamma )P_{k - 1} } } \right)_{\iota } ,\quad (\iota = n + 1, \ldots ,2n) \hfill \\ \end{gathered} \right.$$

$$\overset{\lower0.5em\hbox{$\smash{\scriptscriptstyle\frown}$}}{x}_{k - 1}$$ represents the optimal estimate of the state variables at time k-1, including *SOC*, *Ub*, *U*1, *U*2; $$P_{k - 1}$$ is the state covariance matrix at moment *k*-1;*n* is the number of dimensions of the state variables;γ is the scaling factor.

3. Status variable update:

The obtained *Sigma* points are brought into the nonlinear equation $x_{\iota ,k/k - 1} = {\text{F}} \left( {x_{\iota ,k - 1} ,u_{k} } \right)$ to calculate the predicted values of the mean and covariance of the state variables at moment k:

8 $$x_{\iota ,k/k - 1} = {\text{F}}(x_{\iota ,k - 1} ,u_{k} ),\quad (\iota = 1, \ldots ,2n)$$

9 $$\overset{\lower0.5em\hbox{$\smash{\scriptscriptstyle\frown}$}}{\overline{x}}_{k/k - 1} = \sum\limits_{i = 0}^{2n} {W_{m}^{\iota } } x_{\iota ,k/k - 1} + Q_{k - 1}$$

10 $$P_{k/k - 1} = \sum\limits_{i = 0}^{2n} {W_{c}^{\iota } } \left( {x_{\iota ,k/k - 1} - \overset{\lower0.5em\hbox{$\smash{\scriptscriptstyle\frown}$}}{\overline{x}}_{\iota ,k/k - 1} } \right)\left( {x_{\iota ,k/k - 1} - \overset{\lower0.5em\hbox{$\smash{\scriptscriptstyle\frown}$}}{\overline{x}}_{\iota ,k/k - 1} } \right)^{T} + R_{k - 1}$$

where $Q_{k - 1}$ and $R_{k - 1}$ are the process noise covariance and measurement noise covariance of the previous moment, respectively.

The weight coefficient $W_{m}^{\iota }$ in Eq:

11 $$W_{m}^{0} = \frac{\gamma }{n + \gamma }$$

12 $$W_{m}^{\iota } = \frac{\gamma }{2(n + \gamma )},\quad (\iota = 1, \ldots ,2n)$$

4. Predictive measurement vectors:

The *Sigma* points are transformed using the measurement function to obtain the predicted measurement vector.

13 $$\overset{\lower0.5em\hbox{$\smash{\scriptscriptstyle\frown}$}}{y}_{k/k - 1} = \sum\limits_{i = 0}^{2n} {W_{\iota }^{m} } Y_{\iota ,k/k - 1}$$

14 $$Y_{\iota ,k/k - 1} = g(X_{i,k/k - 1} ,i_{k} ) + R_{k - 1}$$

5. Update state:

Utilize the difference between the measured value and the mean of the predicted measurement vector, along with the Kalman gain, to update the state vector and the state covariance matrix.

Kalman filtering gain update:

15 $$K_{x/k} = P_{xy,k/k - 1} (P_{y,k/k - 1})^{ - 1}$$

New Interest Sequence: The new interest $e_{k}$ represents the difference between the predicted value and the actual observed value.

State variable estimation update:

16 $$\overset{\lower0.5em\hbox{$\smash{\scriptscriptstyle\frown}$}}{x}_{k} = \overset{\lower0.5em\hbox{$\smash{\scriptscriptstyle\frown}$}}{\overline{x}}_{k/k - 1} - K_{k} e_{k}$$

Covariance estimation update:

17 $$P_{k} = \overset{\lower0.5em\hbox{$\smash{\scriptscriptstyle\frown}$}}{P}_{k/k - 1} - k_{k} \overset{\lower0.5em\hbox{$\smash{\scriptscriptstyle\frown}$}}{P}_{y,k} K^{T}_{x/k}$$

### GA-MIUKF algorithm

The GA-MIUKF algorithm combines Genetic Algorithm, multi-innovation Unscented Kalman Filter, and system identification techniques to address state estimation problems in systems with nonlinearity and non-Gaussian characteristics. The GA-MIUKF algorithm utilizes Genetic Algorithm for global search and optimization of system parameters, aiming to achieve more accurate system models and state estimation results. Of particular concern is the impact of the initial SOC value and temperature on the GA-MIUKF algorithm. In literature^15^, research suggests that unknown initial SOC may lead to the algorithm requiring more time to converge when estimating the initial state. The filter may need more measurement data to accurately estimate SOC, potentially making the estimation system more susceptible to noise and measurement errors, resulting in inaccurate estimates and system instability. Temperature is a crucial environmental factor, and temperature variations can alter battery model parameters. Parameters such as internal resistance and open-circuit voltage are typically temperature-dependent. If these parameters are unknown in the estimation algorithm, temperature changes may introduce model uncertainty, affecting the performance of the estimation algorithm^16^.

The GA-MIUKF algorithm for lead–carbon battery State of Charge estimation comprises the following steps:

1. MI-UKF Algorithm Parameter Initialization:Initialize all parameters of the MI-UKF algorithm.

2. Setting System Parameters:

Import the lead-carbon battery model parameters identified using the forgetting factor least squares method into the genetic algorithm. These model parameters are designated as the parameters to be optimized by the genetic algorithm.

Build the system parameter matrix: $$\varepsilon = \left( {\begin{array}{*{20}c} {R_{0} } & {R_{1} } & {R_{2} } & {R_{s} } & {C_{b} } & {C_{1} } & {C_{2} } & {Q_{k} } & {R_{k} } \\ \end{array} } \right)^{T}$$.

3. Global Search and Optimization with Genetic Algorithm:

Employ genetic algorithm for global search and optimization of battery model parameters and noise variance parameters in the MI-UKF algorithm, aiming to enhance estimation accuracy. The genetic algorithm typically involves steps such as initialization, fitness evaluation, selection, crossover, and mutation.

4. Update MI-UKF Algorithm Parameters:

Update the state estimator of the MI-UKF with the optimal system parameters and continue the state estimation process.

5. MI-UKF Algorithm State Estimation for Battery:

Utilize the MI-UKF algorithm for state estimation, comprising prediction and update steps. Initially, based on the state and output equations of the GNL battery model, as well as the current and voltage data, obtain the previous time step's state estimate. Predict the current state values based on the previous time step's state estimate and the battery model. Subsequently, update the state values based on the actual measurement data.

The equation of state and the output equation are given by:

18 $$\left[ {\begin{array}{*{20}c} {\begin{array}{*{20}c} {SOC(k)} \\ {U_{1} (k)} \\ {U_{2} (k)} \\ \end{array} } \\ {U_{b} (k)} \\ \end{array} } \right] = \left[ {\begin{array}{*{20}c} 1 & 0 & 0 & 0 \\ 0 & {e^{{ - \frac{T}{{R_{1} C_{1} }}}} } & 0 & 0 \\ 0 & 0 & {e^{{ - \frac{T}{{R_{2} C_{2} }}}} } & 0 \\ 0 & 0 & 0 & 1 \\ \end{array} } \right]\left( {\begin{array}{*{20}c} {\begin{array}{*{20}c} {SOC(k - 1)} \\ {U_{1} (k - 1)} \\ {U_{2} (k - 1)} \\ \end{array} } \\ {U_{b} (k - 1)} \\ \end{array} } \right) + \left[ {\begin{array}{*{20}c} {\begin{array}{*{20}c} { - \frac{\eta T}{{Q_{old} }}} \\ {R_{1} (1 - e^{{ - \frac{T}{{R_{1} C_{1} }}}} )} \\ {R_{2} (1 - e^{{ - \frac{T}{{R_{2} C_{2} }}}} )} \\ \end{array} } \\ {\frac{T}{{C_{b} }}} \\ \end{array} } \right] \cdot i(k - 1) + Q_{k}$$

19 $$U_{t} (k) = \frac{{R_{s} }}{{R_{s} + R_{0} }}(U_{ocv} [SOC(k)] - U_{1} (k) - U_{2} (k) - U_{b} (k) - R_{0} \times i(k)) + R_{k}$$

**Figure 3:**
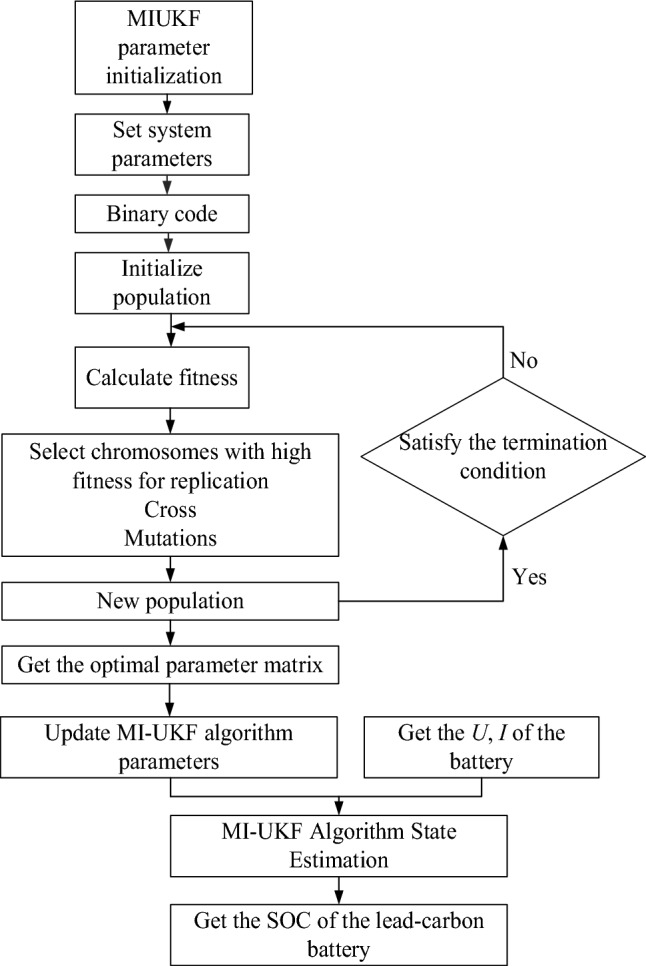
GA-MIUKF algorithm estimates the SOC flow chart of lead–carbon battery.

6. Obtaining the State of Charge (SOC) for Lead–Carbon Battery:

Process the state estimate values to obtain the estimated SOC for the battery. The specific workflow is illustrated in the flowchart shown in Figure 3.

## Results and discussion

### Experiment preparation

**Figure 4:**
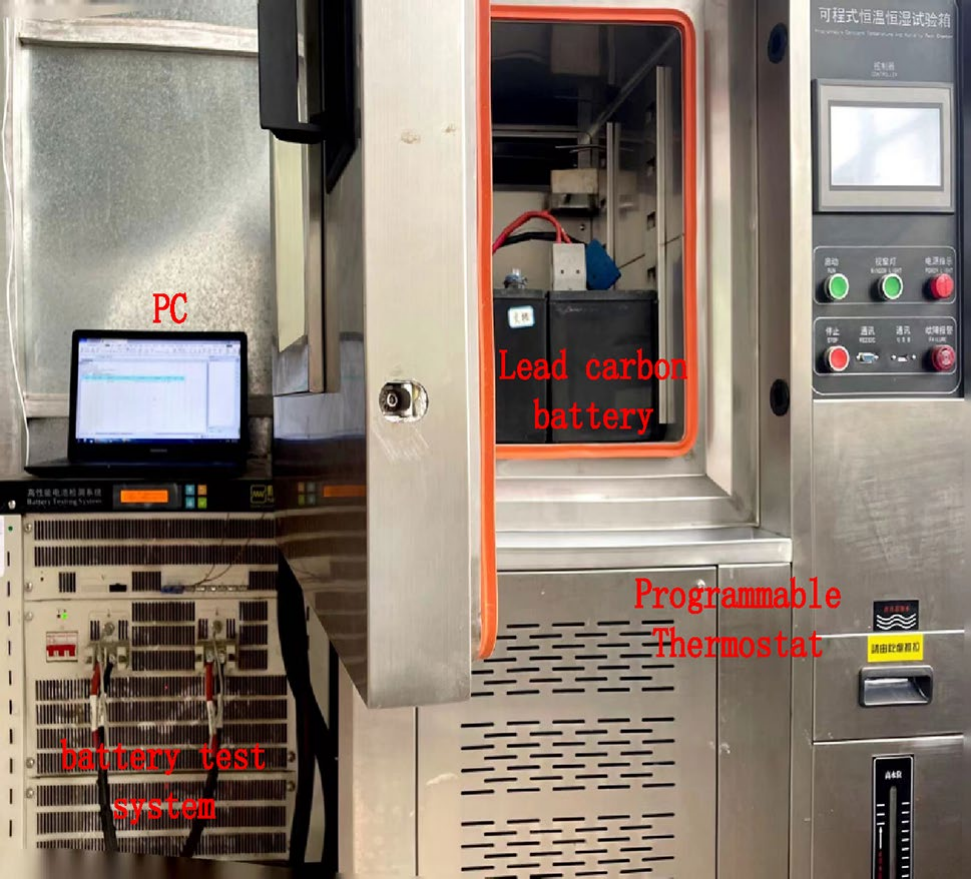
Lead–carbon battery experiment site map.

The study investigates a single 2 V cell with a capacity of 16.67 Ah. This cell is extracted from the 6-GFM-17 lead–carbon battery. The experimental testing instrument employed was the CT-8002 battery testing system. The lead–carbon batteries were placed inside a constant temperature chamber, and the fixture of the battery testing system was attached to the positive and negative terminals of the lead–carbon battery. The battery testing system was controlled by a computer to conduct charging and discharging tests on the lead–carbon battery. The measurement data and dynamic characteristics of the battery were transmitted to the computer through a data acquisition card. The experimental setup of the lead–carbon battery is depicted in Fig. 4.

### Battery parameter identification

#### HPPC testing

For the identification of model parameters, this study employed HPPC (Hybrid Pulse Power Characterization) testing^17^. The HPPC test is capable of identifying the internal parameters of the battery model, providing reliable battery performance data. It possesses advantages such as non-invasiveness, high accuracy, wide frequency range, repeatability, and information richness. The HPPC test allows for the acquisition of the dynamic characteristics and internal parameters of lead–carbon batteries. It can assess the performance of batteries under different SOC and State of Health (SOH) conditions, offering a more comprehensive dataset. Previous research and industry trends have indicated that the HPPC method is an effective choice in this regard. Taking into account experimental conditions, equipment availability, and prior experimental experience, HPPC is the most suitable method for our specific research objectives^18^.

This study utilized a discharge rate of 0.2C for the battery discharge. Choosing a lower discharge rate helps reduce the dynamic response range of the battery during discharge, making the battery’s characteristics easier to observe and analyze. This facilitates a more precise investigation into the power characteristics, voltage response, and other dynamic features of the battery while mitigating the thermal effects and internal pressure variations within the battery^19^.

**Figure 5:**
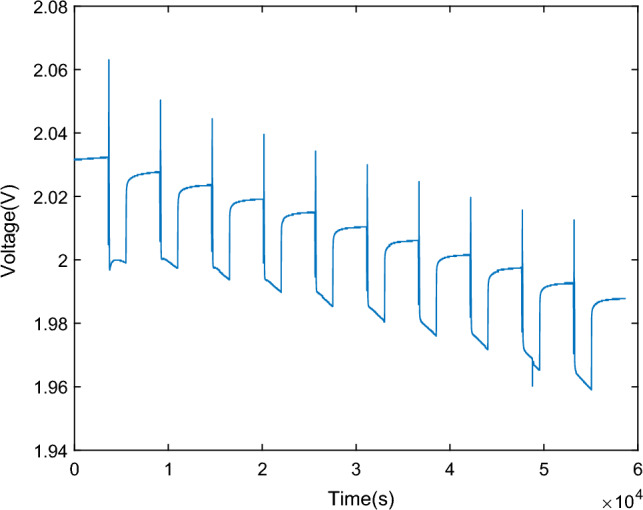
Lead–carbon battery HPPC test terminal voltage curve.

The experimental steps are as follows: Set the temperature of the temperature chamber to 25 °C. ① Rest the fully charged 2 V battery unit for 2 h; ② Discharge at 0.2 C (3.34 A) constant current for 10 s; ③ Stand for 40 s; ④ Charge at 3.34 A constant current for 10 s; ⑤ Stand for 40 s; ⑥ Discharge at 3.34 A current for 30 min; ⑦ Stand for 1 h; ⑧ Cycle steps ②–⑦ for 10 times, sampling time is set to 1 s. The HPPC experiment results are presented in Fig. 5.

**Figure 6:**
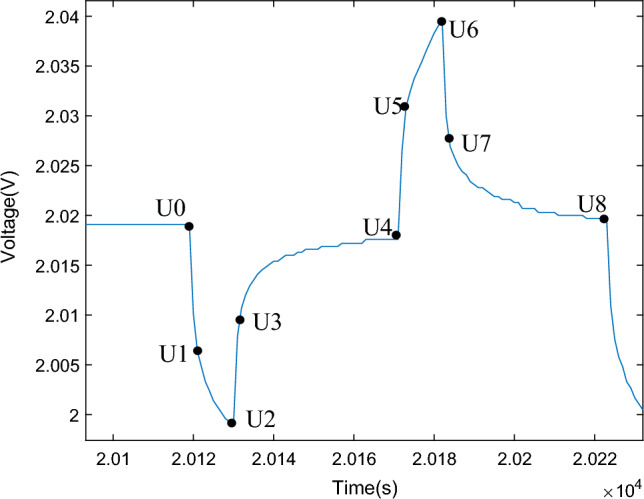
Partial enlarged view of HPPC test voltage.

Figure 6 shows a zoomed-in view of the battery terminal voltage curve during the HPPC experiment. Here, U0 represents the open-circuit voltage. Due to the compliance with Ohm's law by the ohmic resistance and self-discharge resistance, there is an instantaneous change in voltage drop caused by the parallel connection of ohmic resistance and self-discharge resistance at the beginning and end of the current pulse. In the GNL model, the polarization resistance, represented in parallel as a resistor and capacitor, exhibits a hysteresis process in response to the current. Therefore, the voltage drop from U0 to U1 and the voltage rise from U2 to U3 are both caused by the parallel connection of ohmic resistance and self-discharge resistance. The segment from U1 to U2 represents the zero-state response section, while the segment from U3 to U4 represents the zero-input response section.

#### OCV–SOC curve

The OCV curve of the lead–carbon battery is crucial in both equivalent model parameter identification and SOC estimation. Typically, the OCV–SOC relationship function serves as a crucial basis for parameter identification and SOC estimation^20^. This is because the open circuit voltage of the battery is significantly influenced by factors such as battery aging (internal resistance), ambient temperature, and SOC. In this experiment, the influence of ambient temperature and battery aging on open circuit voltage is disregarded.

At a constant temperature of 25 °C, a fully charged 2 V lead–carbon battery cell was allowed to rest for 24 h to achieve internal dynamic equilibrium. Subsequently, a constant current discharge was conducted at a rate of 0.2 C until the cutoff voltage reached 1.8 V, at which point the discharge was halted.

**Figure 7:**
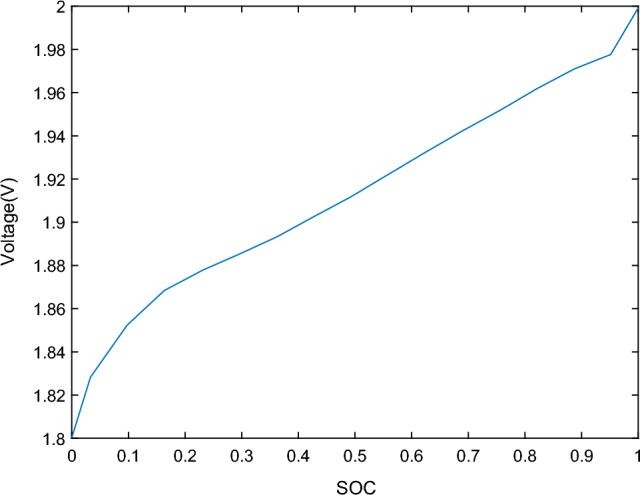
Lead–carbon battery OCV–SOC fitting curve.

According to the fitted curve of the lead–carbon battery OCV–SOC depicted in Fig. 7, the OCV–SOC relationship of the lead–carbon battery demonstrates a generally linear variation. This linear correlation underscores the advantageous performance of lead–carbon batteries and is crucial for parameter identification and SOC estimation. The data for 11 sets of OCV–SOC curves are presented in Table 1.

**Table 1:** The data of the selected 11 sets of OCV–SOC curves.

SOC (%)	100	90	80	70	60	50	40	30	20	10	0
Voltage (V)	2.137	2.035	2.028	2.021	2.014	2.007	2.000	1.994	1.988	1.982	1.975

The functional relationship of OCV–SOC is shown in Eq. 20, which is obtained by fitting using the function toolbox.

20 $$\begin{aligned} Uoc(soc) & = 63.88 \times soc^{7} - 208.3 \times soc^{6} + 267.5 \times soc^{5} - 171.3 \times soc^{4} \\ & \quad + 57.15 \times soc^{3} - 9.389 \times soc^{2} + 0.659 \times soc + 1.965 \\ \end{aligned}$$

#### Parameters of GNL model

When estimating the SOC of the battery, it is necessary to identify the relevant parameters of the battery's second-order GNL equivalent circuit model. In the system parameter identification process, the recursive least squares method with a forgetting factor is employed due to its advantages of not requiring prior statistical knowledge and having low computational complexity. In this study, the parameters *R0*, *R*1, *R*2, *Rs*, *Cb*, *C*1, and *C*2 of the GNL model were identified using the recursive least squares method with a forgetting factor. Ten sets of identification data are listed in Table 2.

**Table 2:** GNL model parameters of lead–carbon battery under different SOC.

SOC	*R*_0_ (Ω)	*R*_1_ (Ω)	*R*_2_ (Ω)	*R*_*s*_ (Ω)	*C*_1_ (F)	*C*_2_ (F)	*C*_*b*_ (F)
1	3.59 × 10^−3^	1.41 × 10^−3^	3.71 × 10^−3^	12,657	140.57	9978.52	6674.40
0.9	3.74 × 10^−3^	2.57 × 10^−3^	2.47 × 10^−3^	14,753	126.35	11,363.62	16,686.00
0.8	3.89 × 10^−3^	1.74 × 10^−3^	1.54 × 10^−3^	11,677	381.15	10,808.07	16,686.00
0.7	3.70 × 10^−3^	2.38 × 10^−3^	1.38 × 10^−3^	12,777	359.45	14,064.94	33,372.00
0.6	3.74 × 10^−3^	2.97 × 10^−3^	2.11 × 10^−3^	11,352	211.34	7335.83	33,372.00
0.5	3.89 × 10^−3^	1.37 × 10^−3^	0.90 × 10^−3^	12,447	346.70	17,646.28	33,372.00
0.4	3.74 × 10^−3^	3.13 × 10^−3^	1.40 × 10^−3^	12,886	130.41	12,574.69	33,372.00
0.3	3.74 × 10^−3^	3.13 × 10^−3^	0.89 × 10^−3^	13,629	144.59	18,654.61	33,372.00
0.2	3.59 × 10^−3^	2.10 × 10^−3^	1.05 × 10^−3^	11,952	299.59	10,902.61	16,686.00
0.1	3.59 × 10^−3^	1.37 × 10^−3^	0.90 × 10^−3^	13,348	349.79	18,014.05	33,372.00

In order to reduce the error of model parameter identification, this paper takes the average value of multiple battery parameters obtained by recursive least squares method as the initial value of parameters, the results are *R*0 = 3.74 × 10^−3^ Ω, *R*1 = 2.31 × 10^−3^ Ω, *R*2 = 1.71 × 10^−3^ Ω, *R*s = 12,646.8 Ω, *C*1 = 237.79 F**,** *C*2 = 12,592.13 F, *C*b = 24,843.60 F.

### UDDS discharge test

**Figure 8:**
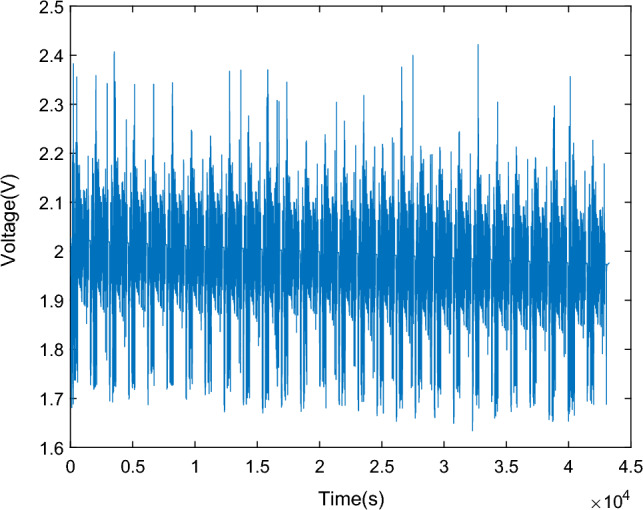
Terminal voltage curve under UDDS working condition.

**Figure 9:**
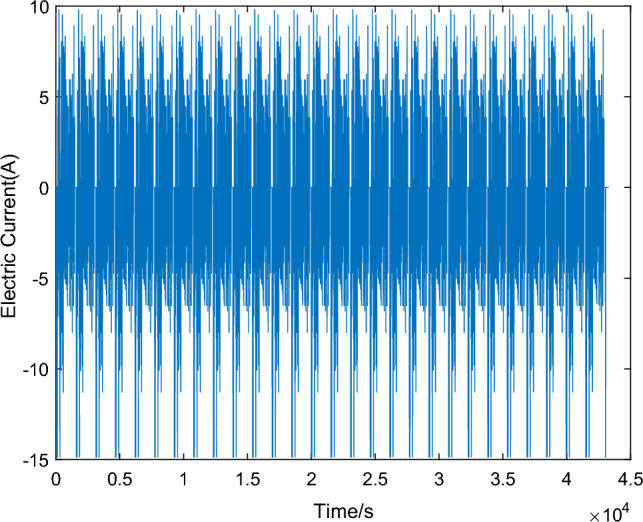
Discharge current curve under UDDS working condition.

**Figure 10:**
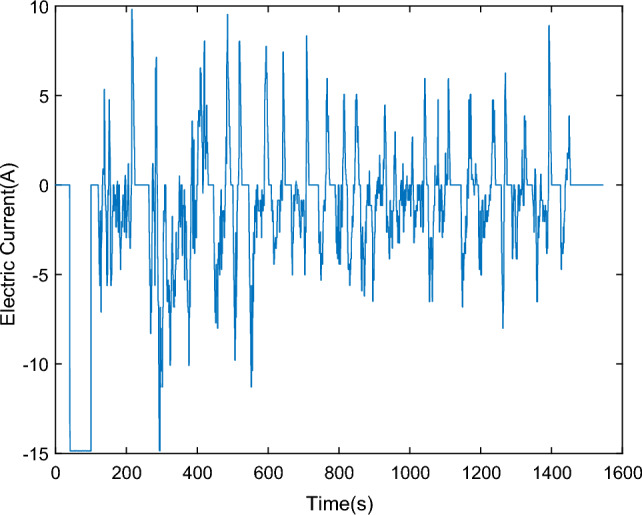
Discharge current curve under single UDDS working condition.

To validate the accuracy of the GA-MIUKF algorithm in SOC estimation, this study adopts the Urban Dynamometer Driving Schedule (UDDS) test proposed by the Society of Automotive Engineers (SAE)^21^. The UDDS test simulates the usage scenarios of lead–carbon batteries under different charge/discharge rates, temperatures, and cycling conditions, allowing for the assessment of their performance, lifespan, and prediction of real-world performance. This is crucial for the selection and application of lead–carbon batteries.The current data for the UDDS cycle is scaled based on literature^22^, utilizing the actual voltage, current, and capacity of lead–carbon batteries. The battery terminal voltage curve is depicted in Fig. 8, and the UDDS cycle current curve is shown in Fig. 9. The current curve for a single cycle is illustrated in Fig. 10. In the validation process, the GA-MIUKF algorithm will be applied to estimate the SOC of the lead–carbon battery under UDDS conditions, and the results will be compared with the actual SOC values to evaluate the accuracy and reliability of the proposed algorithm in real-world driving scenarios.

### Estimating lead–carbon battery SOC with GA-MIUKF algorithm

Based on the algorithm described above, a program is developed to import the UDDS simulated test data and the parameter data identified from the battery model in Table 2 into the GA-MIUKF algorithm, MIUKF algorithm, and UKF algorithm programs. Through online simulation, a comparison is made between UKF and MI-UKF in estimating the SOC of the lead–carbon battery. The performance of the GA-MIUKF algorithm in estimating SOC under UDDS conditions is evaluated.

The SOC estimation for the lead–carbon battery is conducted in a real-world environment, not just in intermediate states. The averaging method, compared to other common methods, provides more flexibility and adaptability, considering changes across the entire SOC range rather than being limited to intermediate states. Therefore, selecting the averaging method as the initial SOC value is more conducive to adapting to the battery's behavior at different SOC levels. For a fully charged battery, the default initial SOC is set to 1.

**Figure 11:**
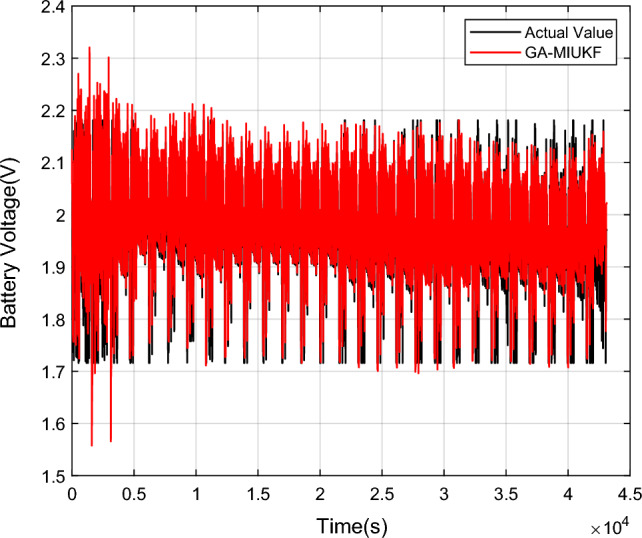
Lead–carbon battery terminal voltage curve estimated by GA-MIUKF.

Figure 11 illustrates the battery terminal voltage curve estimated by the GA-MIUKF algorithm. Under varying current conditions, the captured battery terminal voltage by GA-MIUKF nearly overlaps with the actual battery terminal voltage, demonstrating the tracking and capturing capabilities of the GA-MIUKF algorithm. The SOC estimation results for the lead–carbon battery by GA-MIUKF are observed to be accurate.

**Figure 12:**
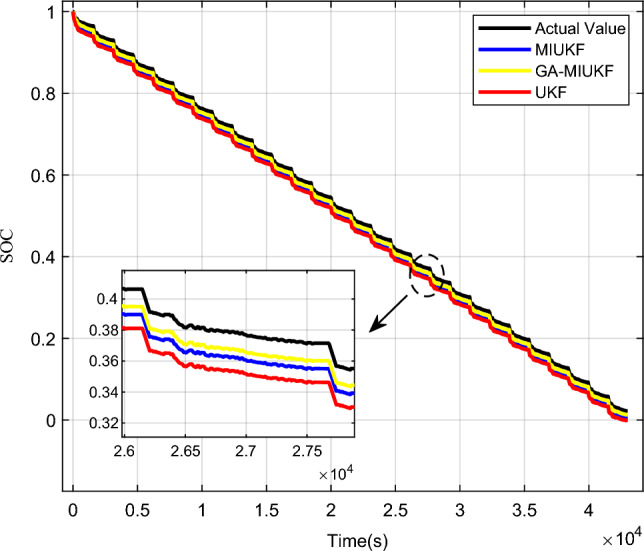
GA-MIUKF, UKF and MIUKF estimate the SOC comparison curves of lead–carbon batteries.

**Figure 13:**
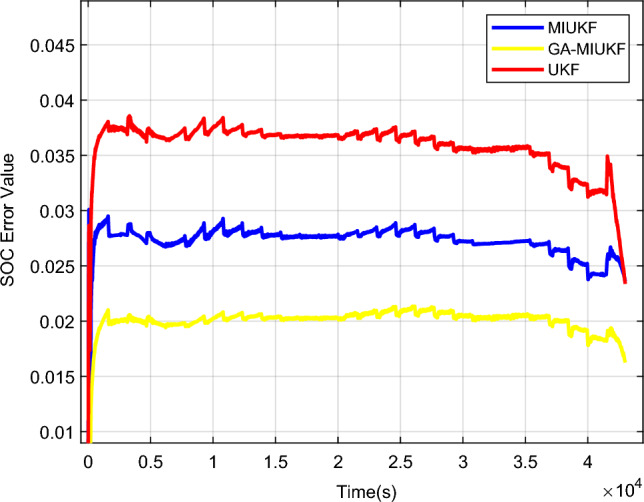
GA-MIUKF, UKF and MIUKF estimate the SOC error curve of lead–carbon batteries.

In this research, the Coulomb counting method was selected for the estimation of the battery’s actual State of Charge (SOC). By measuring the integral current within the battery testing system, real-time acquisition of the cumulative charge allows for the inference of SOC variations. This approach, characterized by its intuitive and efficient nature, synergizes with precise experimental instrument measurements, providing a comprehensive understanding of the battery's dynamic state^23^. Figures 12 and 13 depict the comparative curves and error curves for SOC estimation of lead–carbon batteries under UDDS conditions using GA-MIUKF, UKF, and MIUKF algorithms, respectively.

Utilizing the GA-MIUKF algorithm for State of Charge (SOC) estimation in lead–carbon batteries and comparing the results with those obtained from UKF, MIUKF, and actual measurements, the following outcomes were obtained: the average error in SOC estimation for the UKF algorithm was 3.5%; for the MIUKF algorithm, it was 2.7%; and for the GA-MIUKF algorithm, it was 2.0%. Evidently, in comparison to traditional UKF and MIUKF algorithms, the estimation results derived from the GA-MIUKF algorithm demonstrate higher precision. Therefore, the GA-MIUKF algorithm can effectively enhance the accuracy of SOC estimation in lead–carbon batteries.

## Conclusion

By constructing a second-order GNL equivalent circuit model to simulate lead–carbon batteries and validating it in UDDS simulated operating conditions, this experiment's results demonstrate that using the GA-MIUKF algorithm for estimating lead–carbon battery SOC yields more accurate results, with an average estimation error of only 2.0%. Compared to traditional UKF and MIUKF algorithms, the SOC estimation method based on the GA-MIUKF algorithm better adapts to the characteristics of lead–carbon batteries, significantly improving SOC estimation accuracy and stability. This method holds practical value, providing robust support for state estimation in high-power energy storage batteries, while also offering substantial assistance in battery parameter identification and optimization. Taking into account the diverse computational environments that this algorithm may encounter in practical applications, ranging from powerful computing systems to resource-constrained devices, the challenges associated with its deployment on different platforms are underscored. In future investigations, the focus will be on exploring the adaptability of the algorithm within distinct computational environments, with special consideration given to the performance constraints of resource-constrained devices, such as 8-bit microcontrollers (like Arduino) or 32-bit microcontrollers (like ESP). This dimension of research will contribute to a more comprehensive understanding of the applicability of the GA-MIUKF algorithm, offering practical recommendations for real-world applications.

Future research directions could further explore the application of the GA-MIUKF algorithm in SOC estimation for other types of batteries. Additionally, consideration can be given to applying it in areas such as battery fault diagnosis and health management. This would help expand the algorithm's application scope, enhance its applicability in different battery systems, and provide new directions for further research in the field of battery technology^24^ (Supplementary Information).

### Supplementary Information

Supplementary Information.

## Data Availability

The data employed in this study originates from actual experimental tests. Comprehensive test data for lead and charcoal, inclusive of a data availability statement, is detailed in the paper. Requests for raw data or analysis results can be directed to the corresponding author, facilitating collaboration with fellow researchers.
